# Transcriptomic profiling of immune modulation induced by vitamin D_3_ in the VitDPAS and VitDHiD cohort studies

**DOI:** 10.1038/s41598-025-02495-w

**Published:** 2025-05-19

**Authors:** Emilia Gospodarska, Ranjini Ghosh Dastidar, Julia Jaroslawska, Maciej Rybiński, Marianna Raczyk, Kornelia Tokarczyk-Malesa, Jerzy Romaszko, Carsten Carlberg

**Affiliations:** 1 Institute of Animal Reproduction and Food Research, Polish Academy of Sciences, ul. Trylińskiego 18, 10-683 Olsztyn, Poland; 2 Department of Family Medicine and Infectious Diseases, School of Medicine, Collegium Medicum, University of Warmia and Mazury, Olsztyn, Poland; 3 Institute of Biomedicine, School of Medicine, University of Eastern Finland, Kuopio, Finland

**Keywords:** Vitamin D, Vitamin D intervention studies, Transcriptome, Vitamin D target genes, Vitamin D response index, Inflammatory response, Gene expression, Molecular medicine, Gene regulation in immune cells

## Abstract

The VitDPAS study (NCT06104111) was designed as a medical experiment to assess the in vivo effects of vitamin D on immune responses. This study enrolled 45 healthy individuals from Olsztyn, Poland, who received a body weight-adjusted bolus dose of vitamin D_3_ (1,000 IU/kg). Transcriptome-wide differential gene expression analysis of peripheral blood mononuclear cells, collected before and 24 h after supplementation, identified 758 significantly responsive genes (*p* < 0.05). By correlating individual gene expression changes with alterations in vitamin D status, participants were categorized into three response groups: 17 high responders, 19 mid responders, and 9 low responders. A comparative analysis with the VitDHiD study (NCT03537027), conducted on a Finnish cohort of 25 healthy participants, revealed 232 overlapping target genes, enabling an integrated assessment of vitamin D responsiveness across all 70 individuals. Applying a more stringent statistical threshold (false discovery rate < 0.05) highlighted 26 shared target genes, demonstrating a consistent in vivo response to vitamin D_3_ across both cohorts. The modulation of inflammatory processes, mediated primarily *via* tumor necrosis factor and nuclear factor κB signaling pathways, emerged as a shared effect, highlightening the immunomodulatory potential of vitamin D as a key function of the vitamin in healthy individuals.

**Supplementary Information:**

The online version contains supplementary material available at 10.1038/s41598-025-02495-w.

## Introduction

Vitamin D_3_ is a vital micronutrient that can be synthesized endogenously in human skin when exposed to UV-B light^1^. Both in vitro studies and animal model experiments suggest that vitamin D plays a significant role in modulating the activity of innate and adaptive immunity^2–6^. Observational studies comparing individuals with vitamin D deficiency to those with sufficient levels suggest that adequate vitamin D may provide protective benefits against a range of diseases. These include musculoskeletal disorders such as osteoporosis^7^ and sarcopenia^8^, autoimmune diseases like multiple sclerosis^9^, and certain types of cancer, including colon cancer^10^. In vitamin D deficiency, the underlying issue often relates to insufficient immune modulation^5^. However, conclusive evidence of the beneficial effects of vitamin D supplementation in healthy, non-deficient individuals is lacking. Large-scale intervention studies with thousands of participants over periods up to five years was unable to demonstrate statistically significant primary benefits from vitamin D supplementation, partly due to ethical constraints against leaving control groups vitamin D deficient for extended periods^11,12^.

Vitamin D’s molecular mechanisms involve its nuclear receptor, VDR (vitamin D receptor), which binds the active vitamin D metabolite, 1α,25-dihydroxyvitamin D_3_ (1,25(OH)_2_D_3_)^13,14^, with very high affinity (K_D_ = 0.1 nM). For endocrine signaling, this nuclear hormone is synthesized from 25-hydroxyvitamin D_3_ (25(OH)D_3_) in the kidneys but can also be locally produced in immune and skin cells for para- and autocrine functions^15^. The most abundant and stable metabolite, 25(OH)D_3_, defines vitamin D status in serum^16^, with levels above 30 ng/ml (75 nM) considered sufficient and those below 12 ng/ml (30 nM) indicating severe deficiency^17^.

As a transcription factor, VDR mediates all genomic actions of vitamin D, with 1,25(OH)_2_D_3_ being the exclusive activator of VDR at physiological concentrations^18^. Based on data of the Genotype-Tissue Expression (GTEx) Portal^19^, VDR is expressed in most tissues, excluding the brain, with hundreds of target genes in each tissue^20^, many of which exhibit tissue-specific and individual-specific responsiveness^12,21^. This variation supports the concept of the vitamin D response index, categorizing individuals as high, mid, or low responders to vitamin D supplementation^22^. Notably, low responders may require higher vitamin D dosages to attain optimal physiological benefits^23^.

To investigate vitamin D’s molecular effects in healthy individuals under in vivo conditions, we previously conducted studies such as the VitDbol (NCT02063334, *ClinicalTrials.gov*)^24,25^ and VitDHiD^26,27^, involving healthy cohorts in Kuopio, Finland, who received a single 80,000 IU vitamin D_3_ bolus. In the current study, we expanded on this approach by incorporating the VitDPAS study, which enrolled a cohort of 45 healthy individuals from Olsztyn, Poland. Located more than 1000 km south of Kuopio, the vitamin D winter in Olsztyn is about one month shorter than in Eastern Finland. Transcriptome-wide analysis of peripheral blood mononuclear cells (PBMCs) identified 758 significantly (*p* < 0.05) responsive vitamin D target genes, of which 232 overlapped with targets observed in the VitDHiD study. A core function of these common in vivo vitamin D target genes was the modulation of inflammatory responses.

## Materials and methods

### Sample collection

The VitDPAS intervention study commenced in November 2023 in Olsztyn, Poland. Participants eligible for inclusion were between 20 and 65 years old, with a body mass index (BMI) of 18.5–29.9 kg/m^2^. Exclusion criteria included any disease or infection contracted within the last two weeks before the planned blood draw; severe visual or hearing impairment; gastrointestinal disorders like celiac disease or irritable bowel syndrome; chronic kidney disease; presence of kidney stones; chronic liver diseases; history of pathological bone fractures or fractures due to osteoporosis within the last five years; current diagnosis of cancer; treatment that affects calcium metabolism; diseases with a risk of recurring symptoms, including Parkinson’s disease, post-stroke hemiplegia, epilepsy, recurrent dizziness, and repeated fainting; past or current diseases that may increase serum calcium levels, such as sarcoidosis, lymphoma, and hyperparathyroidism; use of medications that affect 25(OH)D_3_ levels like phenobarbital, carbamazepine, and phenytoin, Paget’s disease of bone (osteitis deformans), arthritis including rheumatoid arthritis, Reiter’s syndrome, and psoriatic arthritis; participation in research studies within the last 4 months, except for observational studies; known infection with HIV, hepatitis, and other infectious diseases; substance abuse like drug dependence, alcoholism, and tobacco smoking; mental illness; homeostasis disorders that may complicate the blood collection process; current pregnancy, breastfeeding, or planned pregnancy. The study protocol was approved by the Ethics Committee of the Olsztyn Chamber of Doctors (#31/2023/VIII).

The study involved a cohort of 45 healthy participants (23 females and 22 males, aged 24–64, BMI 19.3–29.3, Table 1) who received a single vitamin D_3_ bolus of 1,000 IU/kg body mass with breakfast. This bolus equates to a monthly dose of vitamin D_3_, administered only once, whereas other vitamin D interventions, such as the ViDA study^28^, employed a monthly dose of 100,000 IU of vitamin D_3_ over a period of more than two years. Therefore, the dose range of 52,000–110,000 IU vitamin D_3_ used in this study was within safe limits. Blood samples were collected immediately before supplementation (day 0 (d0)) and 24 h after dosing (day 1 (d1)) for serum and PBMC isolation. Based on data collected from previous studies, such as VitDbol^24,25^, a 24-hour post-supplementation period is considered the optimal time point. Serum 25(OH)D_3_ levels were assayed using the electrochemiluminescence binding assay on Cobas Pure Immunoassay Analyzer (Roche) in the analytical laboratory of the Municipal Polyclinical Hospital in Olsztyn, Poland. All participants provided written informed consent, and all experimental procedures adhered to relevant guidelines and regulations.

**Table 1:** VitDPAS study participants.

Participant	Age	Gender	BMI	[25(OH)D_3_] at d0	[25(OH)D_3_] at d1
1	40	F	22.6	26.9	32.3
2	45	F	23.1	19.3	28.6
3	34	F	20.2	16.1	23.7
4	41	F	19.8	38.3	47.3
5	49	F	21.2	29.5	38.0
6	48	F	20.2	24.7	35.2
7	40	F	24.7	21.8	30.4
8	38	F	20.4	19.9	23.6
9	37	F	21.2	26.6	30.0
10	48	F	25.0	18.8	30.0
11	43	F	19.7	21.5	27.8
12	43	F	21.2	34.3	39.9
13	40	F	21.6	47.0	57.5
14	50	F	20.4	35.3	40.1
15	23	F	24.2	30.0	39.3
16	40	F	22.5	31.2	38.7
17	41	F	22.1	27.7	33.4
18	24	F	23.1	16.8	22.2
19	43	F	24.6	28.1	35.5
20	35	F	21.5	20.9	25.0
21	53	F	19.3	54.8	62.6
22	47	F	24.2	14.0	18.6
23	47	F	23.3	21.4	26.0
24	47	M	25.3	41.6	48.7
25	35	M	23.6	12.7	22.1
26	53	M	29.3	30.0	34.7
27	64	M	27.1	41.2	42.7
28	25	M	21.1	24.5	36.4
29	49	M	27.9	23.1	29.1
30	31	M	28.7	15.9	24.2
31	27	M	28.6	14.3	20.6
32	47	M	29.6	16.3	21.0
33	40	M	24.0	55.9	57.8
34	37	M	23.4	33.7	41.9
35	24	M	21.2	25.7	31.4
36	25	M	20.3	25.6	30.5
37	37	M	19.3	15.3	20.7
38	39	M	27.6	32.4	34.7
39	34	M	27.5	41.7	45.1
40	47	M	29.5	26.6	32.7
41	25	M	20.6	24.9	29.7
42	31	M	24.9	9.0	15,2
43	38	M	27.2	17.4	22.5
44	38	M	28.9	11.4	19.9
45	42	M	28.3	28.6	32.1

Age, gender, BMI and 25(OH)D_3_ serum levels (in ng/ml) at d0 and d1 are indicated.

### PBMC isolation

PBMCs from the 45 participants of the VitDPAS study were promptly processed without any in vitro culture. Following the collection of 8 ml of peripheral blood on d0 and d1, PBMCs were isolated within one hour using Vacutainer CPT Cell Preparation Tubes with sodium citrate (Becton Dickinson), following the manufacturer’s protocol. After isolation, cells were washed with phosphate-buffered saline, aliquoted at a concentration of 4 million cells per ml, and preserved at -80 °C for future RNA isolation.

### Transcriptome analysis

Total RNA was extracted from PBMCs using the High Pure RNA Isolation Kit (Roche) following the manufacturer’s instructions. RNA quality was assessed using an Agilent Tapestation, and library preparation was conducted after rRNA depletion with kits and protocols from New England Biolabs. RNA-seq libraries were sequenced at a read length of 75 bp on a NextSeq2000 system (Illumina) according to standard protocols at the EMBL Gene Core (Heidelberg, Germany). Quality control of the sequencing files was performed using FastQC (version 0.11.9, http://www.bioinformatics.babraham.ac.uk/projects/fastqc) (Supplementary Table S1 online). Reads were aligned to the GRCh38 reference genome (Ensembl version 111.38) using the STAR aligner^29^ (version 2.7.2a), and quantification was carried out with FeatureCounts^30^ (version 2.0.1) using default parameters. To standardize gene nomenclature across all datasets, Human Gene Nomenclature Committee (HGNC) symbols were updated using the R package HGNChelper (version 0.8.1, https://CRAN.R-project.org/package=HGNChelper). Annotation, including gene identifiers, descriptions, genomic locations, and biotypes, was added *via* the Ensembl database (release 100) using the R package BiomaRt^31^ (version 2.42.1). Entrez Gene identifiers were incorporated using the R package org.Hs.eg.db (version 3.10.0), and any incomplete mappings for target genes were manually verified and retrieved from NCBI (http://www.ncbi.nlm.nih.gov/home/genes). Genes lacking genomic position information or those mitochondrially encoded were excluded from the analysis. This study focused solely on protein-coding genes.

### Differential gene expression analysis

Differential gene expression analysis was conducted in R (version 4.3.1) on MacOS 13 (Ventura) using the *EdgeR* package^32^ (version 4.0.16) for robust assessment. To reduce transcriptional noise associated with non-coding genes, the analysis focused on 19,287 protein-coding genes. Read counts were normalized to counts per million (CPM) to account for library size differences. Low-expression genes (CPM < 10) were filtered out using the FilterByExpr() function, minimizing the multiple testing burden and optimizing statistical accuracy within the *EdgeR* framework. Following filtering, library sizes were recalculated, and the trimmed mean of M-values normalization was applied. The transcriptome data structure was explored using multidimensional scaling (MDS) *via EdgeR*’s plotMDS() function, where distances approximate typical log_2_ fold changes (FC) between samples. These distances were calculated as the root mean square deviation (Euclidean distance) of log_2_FC values for genes showing significant changes (p-value < 0.05 or Benjamini-Hochberg adjusted p-value = FDR (false discovery rate) < 0.05)) post-vitamin D_3_ supplementation. Mean-Difference (MA) plots were generated with *vizzy* (version 1.0.0, https://github.com/ATpoint/vizzy). Differential expression for each gene was determined through the generalized linear model quasi-likelihood (GLM-QL) pipeline. A trended negative binomial dispersion estimate was calculated using the Cox-Reid profile-adjusted likelihood method. This estimate, along with empirical Bayes-moderated quasi-likelihood gene-wise dispersion estimates^33^, was employed for GLM fitting, with empirical Bayes shrinkage robustified against outlier dispersions, as recommended. To identify differentially expressed genes with substantial changes, the glmTreat approach^33^ was applied, setting a threshold for absolute log_2_FC > 0.25 (Supplementary Table S2 online).

To integrate the gene expression data from the VitDPAS and VitDHiD cohorts, matched common gene identifiers across both datasets were identified, ensuring that only shared genes were included in the combined dataset. To account for differences in sequencing depth, the expression values were normalized using CPM, which adjusts raw counts accordingly. Additionally, a scaling step was applied to harmonize the data across time points (d0 and d1). This involved calculating scaling factors based on the average gene expression levels of both cohorts and adjusting the data to minimize technical discrepancies, thereby facilitating a more accurate comparison.

### Classification of study participants

To classify participants as high, mid, or low responders, the average absolute log_2_FC values of all in vivo vitamin D target genes (or relevant subsets) for each individual (Supplementary Table S2 online) was plotted against the changes in total 25(OH)D_3_ concentrations (d1/d0) (Table 1). Based on prior experience with the vitamin D intervention studies VitDmet^34^ and VitDbol^24,25^, plotting the ratio of 25(OH)D_3_ values proves to be more effective than using their delta. A trendline was drawn through the 1/0 point to divide the plot, and the orthogonal distance of each data point from this line was calculated. These distances were then ranked in descending order, and clustering of participants into three response groups was performed using the *K-means* algorithm (KMeans function from the sklearn.cluster library, available at https://pypi.org/project/scikit-learn/*).*

### Functional characterization of vitamin D target genes

The STRING database (http://string-db.org/)^35^ was used to confirm and visualize the physical and functional interactions among vitamin D target genes. In this network, each node represents a protein, while edges between nodes indicate associations inferred from a combination of experimental, computational, and literature-derived data. Functional annotation of the genes, with an emphasis on their primary roles, was derived from databases like Human Protein Atlas (http://www.proteinatlas.org) and GeneCards (http://www.genecards.org).

## Results

### The VitDPAS intervention trial

The VitDPAS study design is detailed in Supplementary Figure S1A online. The study began by administering a weight-adjusted vitamin D_3_ bolus (1,000 IU/kg) to 45 healthy participants (23 females, 22 males; mean age: 39.6 years; mean BMI: 23.8, Supplementary Figure S1B online). Blood samples were collected immediately before supplementation (d0) and 24 h afterward (d1). Baseline serum 25(OH)D_3_ levels at d0 ranged from 9.0 to 55.9 ng/ml, with an average of 26.5 ng/ml, while levels at d1 increased to a range of 18.6 to 62.6 ng/ml, with an average of 32.9 ng/ml (Table 1). Thus, the vitamin D_3_ bolus resulted in a significant average increase in vitamin D status by 6.4 ng/ml, or 29% (*p* = 3.8 × 10^− 23^, paired T-test, Supplementary Figure S1C online). In summary, a single, weight-adjusted vitamin D_3_ bolus was sufficient to elevate the vast majority of VitDPAS participants to a level of vitamin D sufficiency.

### Vitamin D-induced transcriptomic response in VitDPAS participants

**Fig. 1:**
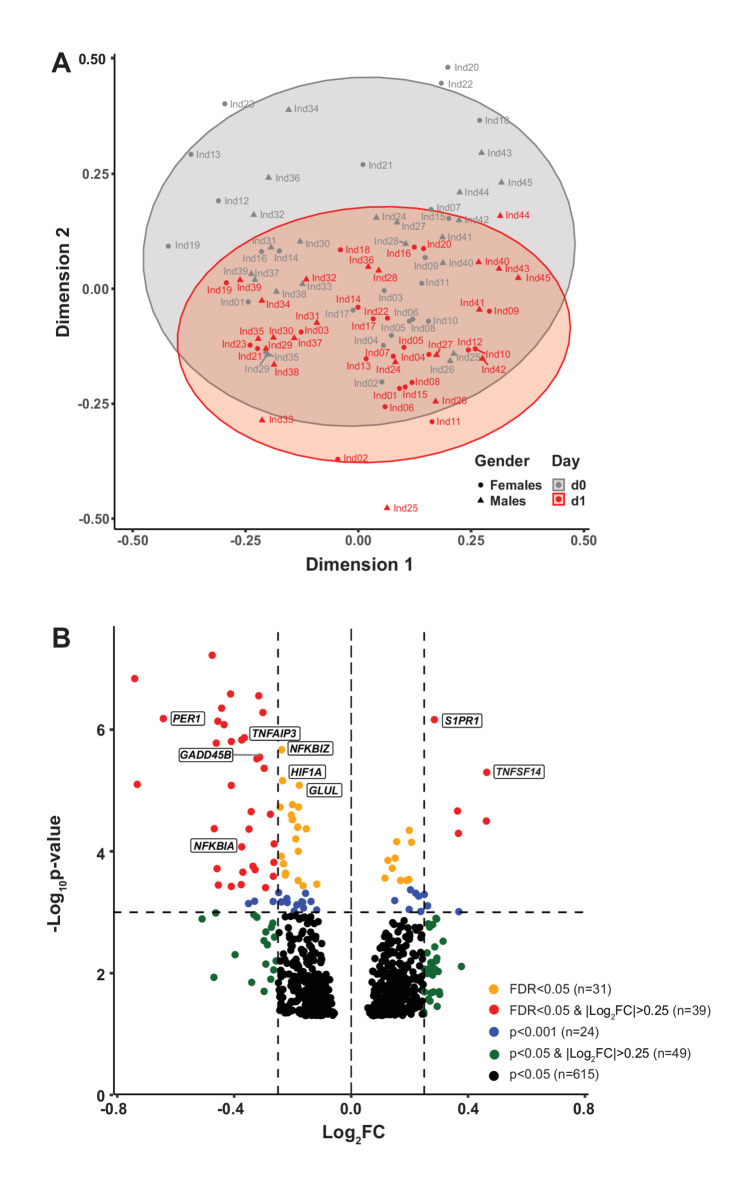
Transcriptome-wide analysis of vitamin D_3_ bolus supplementation in the VitDPAS cohort. (**A**) The quality of transcriptome datasets (of 758 target genes) from the 45 VitDPAS participants at d0 and d1 was assessed using dimensionality reduction techniques, visualized through a MDS plot. (**B**) A Volcano plot was employed to illustrate the impact of vitamin D_3_ supplementation on the expression of 758 significantly regulated genes (*p* < 0.05). Selected target genes are highlighted.

Differential gene expression analysis of RNA-seq data at d1 compared to baseline (d0) revealed that vitamin D_3_ bolus supplementation significantly impacted the transcriptome of the 45 VitDPAS participants. Principal component analysis, visualized in an MDS plot, showed a notable shift, with d1 data points averaging below the respective d0 points along dimension 2 (Fig. 1A). Out of 8,937 protein-coding genes expressed in PBMCs across all participants (defined as mean CPM > 10 for d0 and d1), 758 genes were significantly (*p* < 0.05) regulated by vitamin D (Figure S2). Of these, 369 were upregulated and 389 downregulated (Supplementary Table S2 online, Supplementary Figure S2 online). Applying an additional threshold of absolute log_2_FC > 0.25 narrowed this list to 94 vitamin D target genes, including 39 upregulated and 55 downregulated. A stricter criterion of FDR < 0.05 identified 70 genes, with 15 upregulated and 55 downregulated, and 39 of these also meeting the absolute log_2_FC > 0.25 threshold (5 upregulated and 34 downregulated) (Fig. 1B). Taken together, vitamin D_3_ bolus supplementation significantly modulates the transcriptome of circulating immune cells in VitDPAS participants.

### Segregation of VitDPAS study participants by vitamin D responsiveness

**Fig. 2:**
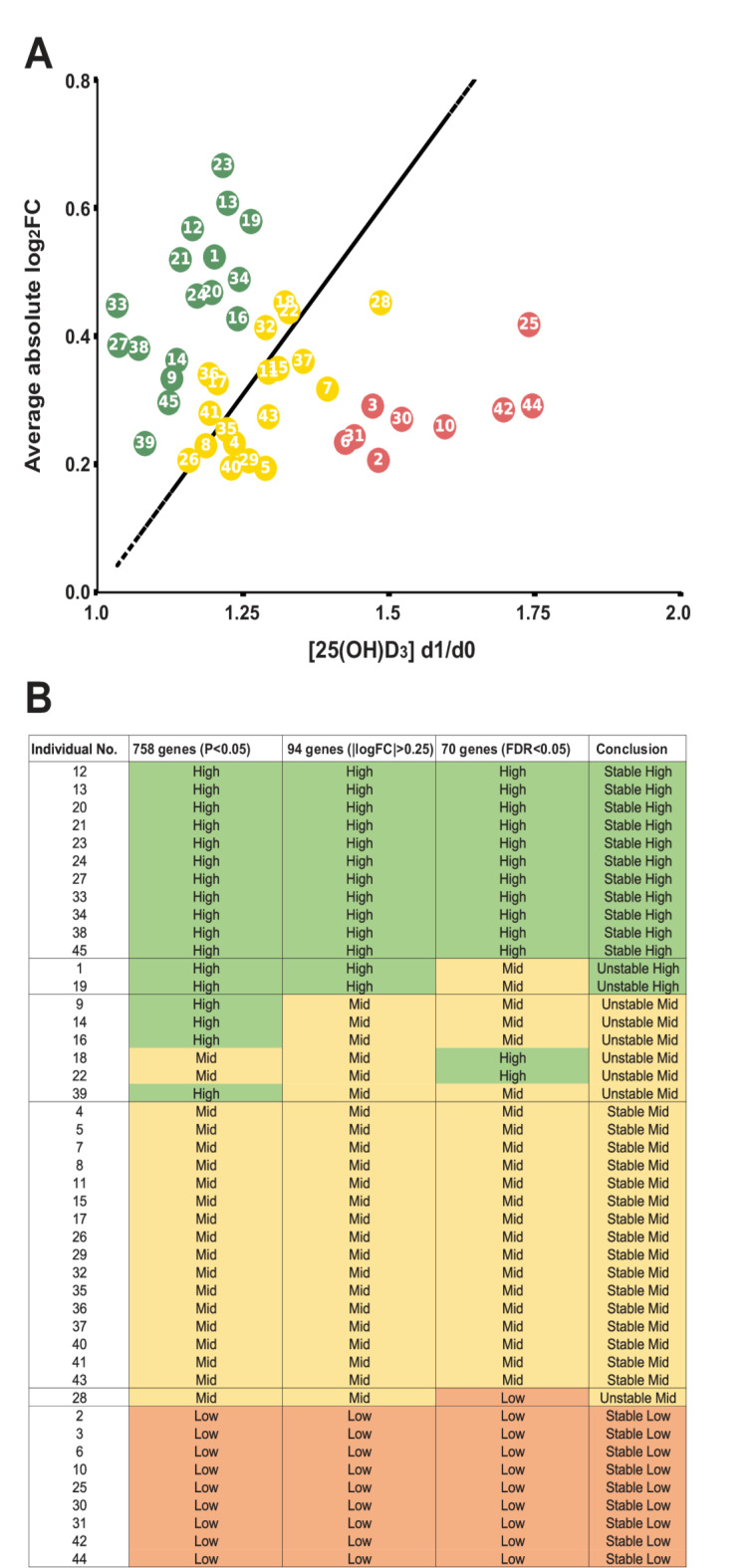
Classification of VitDPAS study participants. (**A**) The 45 VitDPAS participants were classified into high (green), mid (yellow), and low (red) responders based on the expression profiles of all 758 in vivo vitamin D target genes. (**B**) Comparison of classification methods utilizing various subsets of vitamin D target genes to evaluate the robustness of the segregation approach.

To evaluate the vitamin D responsiveness among the 45 VitDPAS participants, each individual’s average absolute log_2_FC of all 758 vitamin D target genes was plotted against the ratio of their vitamin D status at d1 relative to baseline (d0) (Fig. 2A). This analysis revealed a spectrum of responsiveness, with high responders clustering at the upper left of the plot (green), mid responders near the trendline (yellow), and low responders at the lower right (red). The distance from the trendline was proportional to each participant’s vitamin D response index, which showed a strong negative correlation (-0.82, Pearson correlation, *p* = 3.7 × 10^− 12^, paired T-test) with the d1/d0 vitamin D status ratio. This response index correlated less strongly with baseline vitamin D status (0.61, *p* = 0.0000089, Supplementary Figure S3A online), and showed no significant correlation with age (0.23, *p* = 0.12, Supplementary Figure S3B online) or BMI (-0.08, *p* = 0.58, Supplementary Figure S3C online) of the individuals. *K-means* clustering further classified the participants into 9 low responders, 19 mid responders, and 17 high responders (Supplementary Figure S4 online). When the analysis was limited to the 94 genes with an absolute log_2_FC > 0.25, the results were similar; however, participants #9, 14, 16, and 39 were reclassified as mid responders (Fig. 2B). Narrowing the gene list further to 70 targets with FDR < 0.05, individuals #1, 9, 14, 16, 19, and 39 were reclassified as mid responders, while participants #18 and #22 were newly classified as high responders, and #28 as a low responder. Consequently, participants #1 and #19 appear as unstable high responders, while #9, 14, 16, 18, 22, 28, and 39 are unstable mid responders. In summary, VitDPAS participants were stratified into high, mid, and low responders to vitamin D_3_ supplementation.

### Joint classification of VitDPAS and vitdhid participants

**Fig. 3:**
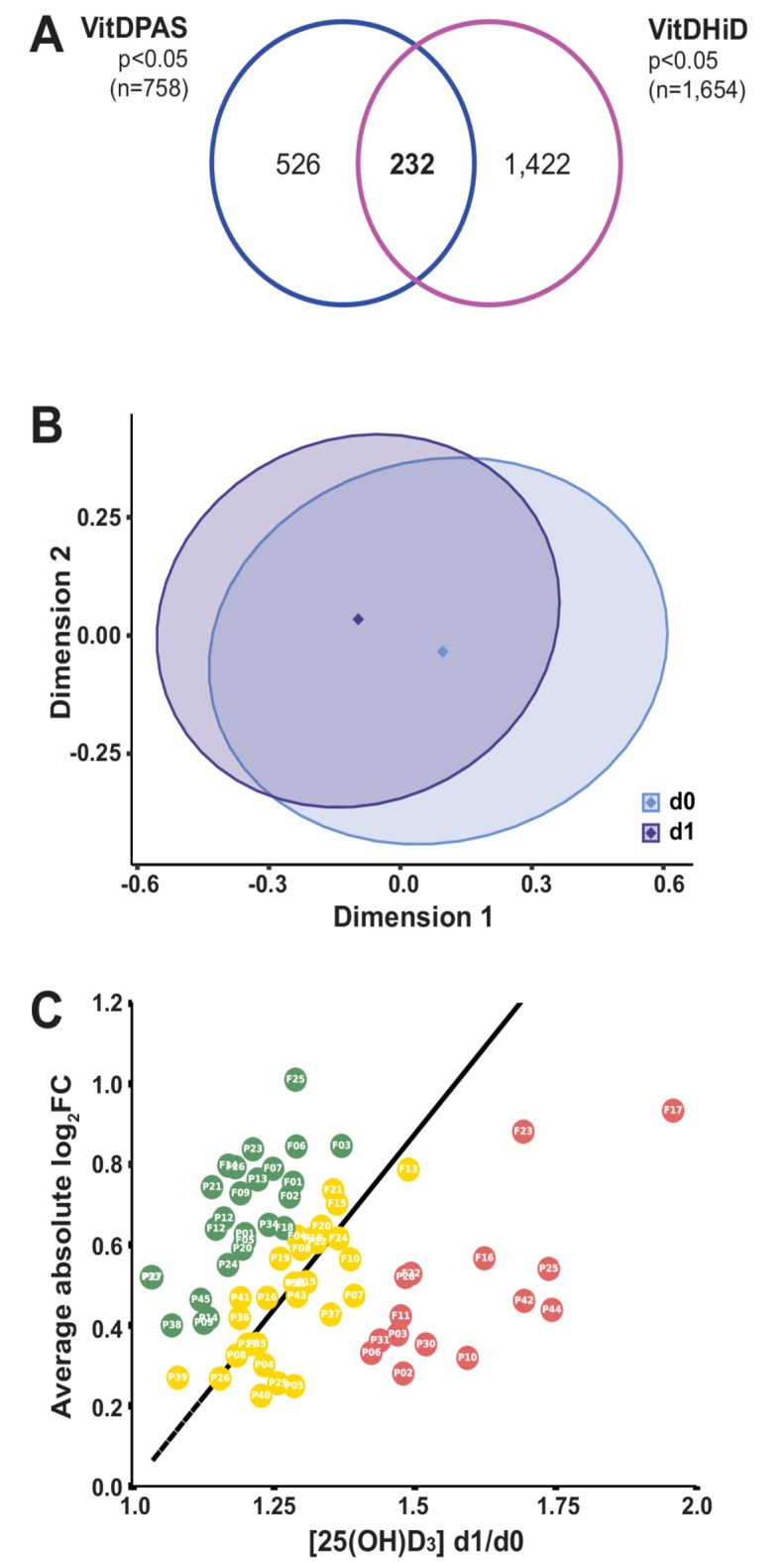
Overlap and classification of vitamin D target genes between VitDPAS and VitDHiD studies. (**A**) A Venn diagram highlights the intersection of 758 significant (*p* < 0.05) in vivo vitamin D target genes identified in the VitDPAS study and 1,654 target genes from the VitDHiD study. (**B**) The quality of transcriptome datasets for the 232 shared target genes at d0 and d1 was assessed using dimensionality reduction, depicted in a MDS plot. (**C**) Based on the expression profiles of the 232 common target genes, the 70 participants across both studies were categorized into high (green), mid (yellow), and low (red) vitamin D responders.

Comparison of the 758 in vivo vitamin D target genes from the VitDPAS study with the 1,654 targets identified in the VitDHiD study (*p* < 0.05) revealed 232 shared genes (Fig. 3A). The two studies, which both investigated healthy individuals (VitDHiD with 25 participants from Finland), were highly comparable and analyzed using identical methods. The combined transcriptome datasets from all 70 participants were normalized and analyzed using principal component analysis *via* MDS plots. Based on the 232 common target genes, on average, d1 datasets clustered to the left of d0 datasets along the first dimension, suggesting that vitamin D_3_ supplementation consistently impacted the PBMC transcriptome across both cohorts (Fig. 3B). Segregation of the 70 individuals by vitamin D responsiveness classified them into 22 high responders, 34 mid responders, and 14 low responders (Fig. 3C). Four VitDPAS participants were reclassified based on this joint analysis: participant #19 shifted from unstable high to mid responder, #16 and #39 from unstable mid to mid responders, and #28 from unstable mid to low responder. Similarly, four VitDHiD participants were reclassified (three from mid to high responders and one from mid to low responder)^27^. For the remaining 41 VitDPAS and 21 VitDHiD participants (88.6% overall), the joint analysis produced results consistent with each individual study’s classification. In summary, this combined analysis of the VitDPAS and VitDHiD vitamin D intervention studies identified 232 common target genes and yielded consistent response classifications for 62 of the 70 participants.

### Functional impact of joint VitDPAS and vitdhid target genes

**Fig. 4:**
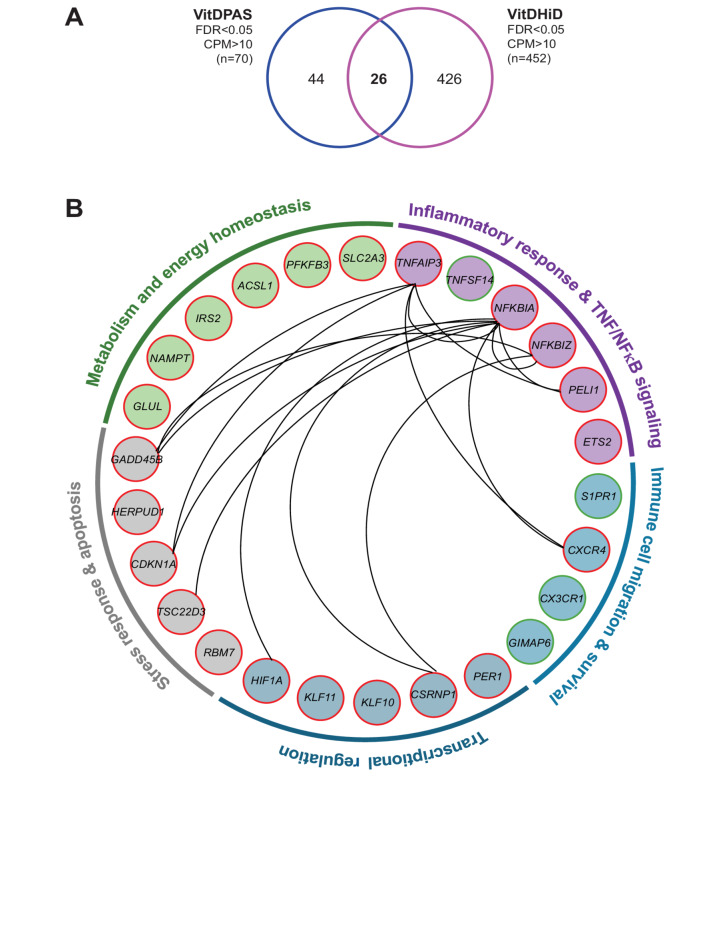
Overlap and functional classification of vitamin D target genes between VitDPAS and VitDHiD studies. (**A**) A Venn diagram illustrates the overlap between 70 highly significant (FDR < 0.05) in vivo vitamin D target genes identified in the VitDPAS study and 452 target genes from the VitDHiD study. (**B**) The 26 shared vitamin D target genes are grouped into distinct functional categories. Gene interactions, derived from the STRING database (Supplementary Figure S8 online), are depicted with connecting lines. Upregulated genes are shown in green, while downregulated genes are represented in red.

A stringent comparison (FDR < 0.05) between 70 vitamin D target genes identified in the VitDPAS study and 452 targets from the VitDHiD study^27^ revealed 26 shared genes (Fig. 4A). Notably, of these common genes, only 4 were upregulated by vitamin D_3_ supplementation, while 22 were downregulated (Supplementary Figure S5 online). The 26 genes were validated by two different approaches. A repeated vitamin D_3_ bolus supplementation with the same study participants indicated for 80–90% of genes a comparable regulation on the RNA level (Supplementary Figure S6 online). Moreover, an ATAC-seq (assay for transposase accessible chromatin with high-throughput sequencing) analysis for accessible chromatin at TSS (transcription start site) regions confirmed for all downregulated genes a decrease in accessibility (Supplementary Figure S7 online).

The STRING database was utilized to explore potential functional relationships among the proteins encoded by the 26 shared genes (Supplementary Figure S8 online). This analysis, combined with a review of publicly available databases such as Human Protein Atlas and GeneCards, facilitated the classification of these genes/proteins into five distinct functional categories: (i) “inflammatory response and TNF (tumor necrosis factor)/NFκB (nuclear factor κB) signaling includes *TNFAIP3* (TNF alpha-induced protein 3), *TNFSF14* (TNF superfamily member 14), *NFKBIA* (NFκB inhibitor alpha), *NFKBIZ* (NFκB inhibitor zeta), *PELI1* (pellino E3 ubiquitin protein ligase 1), and *ETS2* (ETS proto-oncogene 2, transcription factor); (ii) “immune cell migration and survival” comprises *S1PR1* (sphingosine-1-phosphate receptor 1), *CXCR4* (C-X-C motif chemokine receptor 4), *CX3CR1* (C-X3-C motif chemokine receptor 1), and *GIMAP6* (GTPase, IMAP family member 6); (iii) “transcriptional regulation” contains *PER1* (period circadian regulator 1), *CSRNP1* (cysteine and serine-rich nuclear protein 1), *KLF10* (KLF transcription factor 10), *KLF11*, and *HIF1A* (hypoxia-inducible factor 1 subunit alpha); (iv) “stress response and apoptosis” consists of *RBM7* (RNA binding motif protein 7), *TSC22D3* (TSC22 domain family member 3), *CDKN1A* (cyclin-dependent kinase inhibitor 1A), *HERPUD1* (homocysteine-inducible ER protein with ubiquitin-like domain 1), and *GADD45B* (growth arrest and DNA damage-inducible beta); (v) “metabolism and energy homeostasis” embraces *GLUL* (glutamate-ammonia ligase), *NAMPT* (nicotinamide phosphoribosyltransferase), *IRS2* (insulin receptor substrate 2), *ACSL1* (acyl-CoA synthetase long-chain family member 1), *PFKFB3* (6-phosphofructo-2-kinase/fructose-2,6-biphosphatase 3), and *SLC2A3* (solute carrier family 2 member 3) (Fig. 4B). Interestingly, based on EnrichR^36^ analysis of the 232 common target genes between VitDPAS and VitDHiD (Fig. 3A), TNF signaling is the top ranking pathway in the database KEGG (Kyoto Encyclopedia of Genes and Genomes)^37^. Furthermore, according to STRING analysis (Supplementary Figure S8 online), NFKBIA, HIF1A, TNFAIP3, GADD45B, CXCR4, NFKBIZ, CDKN1A, and CSRNP1 represent the most well-characterized core within this protein set. NFKBIA, NFKBIZ, and TNFAIP3 function downstream of stress sensors, including GADD45D (responsive to DNA damage), CDKN1A (cell cycle regulation), HIF1A (hypoxia response), and CXCR4 (immune activation) (Fig. 4B). Interestingly, in the majority of healthy individuals across both studies (84.3%), the genes encoding these proteins are downregulated in response to vitamin D_3_ supplementation, a trend that also holds for the average expression of the full set of 26 genes (Supplementary Figure S5 online). However, in a subset of 7 individuals, this gene set is upregulated, indicating an opposite effect of vitamin D_3_ for these persons. In summary, the functional classification of 26 common highly significant target genes emphasizes a range of biological roles influenced by vitamin D_3_ supplementation. A central outcome of this regulation is the downregulation of vitamin D target genes involved in inflammatory responses, primarily through TNF and NFκB signaling pathways.

## Discussion

The primary aim of this study was to validate and expand the concept of the vitamin D response index in a European cohort distinct from the one where it was originally developed^22^. Unlike other studies that examined gene expression changes following vitamin D_3_ supplementation over an 8-week period^38^, the VitDPAS study employed a different approach: administering a single vitamin D_3_ bolus and taking a second measurement just one day later. This methodology facilitates direct comparison with in vitro studies, which traditionally use 24-hour stimulations with 1,25(OH)₂D₃^39^. By bridging these experimental paradigms, the VitDPAS findings offer valuable insights into vitamin D’s gene-regulatory effects on immune function in healthy individuals, potentially advancing our understanding in this area.

The VitDPAS study described here, conducted with a Polish cohort, represents an advanced extension of our previous VitDHiD study^26^ performed in Kuopio, Finland. Both studies included healthy participants; however, the 45 VitDPAS participants were, on average, 12.1 years older and had a 1.1 kg/m^2^ higher BMI compared to the 25 VitDHiD participants. Despite these differences, the distribution of high, mid, and low vitamin D responders was similar across both studies, with rates of 37.8%, 42.2%, and 20% in VitDPAS, closely aligning with 36%, 48%, and 16% in VitDHiD, respectively. Interestingly, when combining data from the 70 participants of both studies, the overall response rates were 40% high responders, 38.6% mid responders, and 21.4% low responders. For comparison, a similarly designed study conducted with 50 male and 50 female participants in Jeddah, Saudi Arabia (21°N)^40,41^, identified 22% high responders, 39% mid responders, and 39% low responders^42^. This indicates that the proportion of low responders in a population living closer to the equator is nearly double that observed in Northern or Central European populations.

The regions of the two vitamin D intervention studies, Olsztyn (53°N) and Kuopio (63°N), experience extended “vitamin D winters”^43,44^ lasting approximately 4 and 5 months, respectively. These are periods during which endogenous vitamin D_3_ production is not possible. This is particularly critical for individuals classified as low vitamin D responders, who require adequate vitamin D_3_ supplementation during the winter months to prevent health issues associated with vitamin D deficiency^45^. Although, daily vitamin D_3_ supplementation of up to 4000 IU is considered safe^46^, we recommend VitDPAS participants to adjust their daily intake during the winter months according to their body weight, ensuring it does not exceed 40 IU of vitamin D_3_/kg. The VitDPAS study is currently ongoing, with participants being supplemented and monitored over a total duration of two years.

Previous reports have highlighted significant interindividual variability in the sets of in vivo vitamin D target genes^26^. The transcriptome analysis of the 45 participants in the VitDPAS study supports this finding. Out of the 8,397 protein-coding genes expressed at a reasonable level (CPM > 10) in the PBMCs of all participants, 758 (9.0%) exhibited a significant (*p* < 0.05) response to vitamin D. However, only 232 of these vitamin D target genes (30.6%) overlapped with those identified in the VitDHiD study. While the overlap increases to 37.1% when using more stringent statistical criteria (FDR < 0.05), this still suggests that only a small subset of in vivo vitamin D targets (26 genes) is consistently identified across both studies with high confidence.

An intriguing observation from the VitDPAS study is that, although the initial list of 758 vitamin D target genes included an approximately equal number of upregulated and downregulated genes, only 4 of the 26 genes in the final shortlist were upregulated. This finding suggests that the most critical targets of vitamin D in healthy individuals are more likely to be downregulated than elevated. This aligns with previous findings from the VitDHiD study^47^, which indicated that vitamin D-triggered pathways are predominantly downregulated or maintained in equilibrium, rather than being actively upregulated.

In a previous study utilizing VitDHiD data, the genes *NFKBIA*, *NFKBIZ*, and *TNFAIP3* were identified as top-ranking players in eight signaling pathways related to innate immunity^48^. Interestingly, the downregulation of NFKBIA and NFKBIZ, both inhibitors of NFκB, promotes the translocation of the transcription factor NFκB from the cytoplasm to the nucleus, thereby activating multiple proinflammatory target genes. Moreover, downregulation of TNFAIP3, an upstream inhibitor of TNF-mediated inflammatory pathways, may further amplify the inflammatory response. These findings suggest that vitamin D enhances the sensitivity of inflammatory signaling pathways to external triggers, thereby facilitating a more efficient response when necessary.

In conclusion, the VitDPAS study validated and expanded upon the principles of the vitamin D response index. It underscored the need for special attention to low vitamin D responders within this Polish cohort, ensuring adequate vitamin D_3_ supplementation during the winter months. The combined analysis of the VitDPAS and VitDHiD studies identified 26 high-confidence vitamin D target genes, whose core function is the regulation of inflammatory responses in response to external stress sensors.

## Electronic supplementary material

Below is the link to the electronic supplementary material.

Supplementary Material 1

Supplementary Material 2

Supplementary Material 3

## Data Availability

Fastq files of the raw data can be found at Gene Expression Omnibus (GEO, http://www.ncbi.nlm.nih.gov/geo) with accession number GSE278885.

## Abbreviations

1,25(OH)_2_D_3_: 1α,25-dihydroxyvitamin D_3_
25(OH)D_3_: 25-hydroxyvitamin D_3_
ACSL1: Acyl-CoA synthetase long chain family member 1
ATAC-seq: assay for transposase accessible chromatin with high-throughput sequencing
BMI: Body mass index
CDKN1A: Cyclin dependent kinase inhibitor 1 A
CPM: Counts per million
CSRNP1: cysteine and serine rich nuclear protein 1
CX3CR1: C-X3-C motif chemokine receptor 1
CXCR4: C-X-C motif chemokine receptor 4
ETS2: ETS proto-oncogene 2, transcription factor
FC: Fold change
FDR: False discovery rate
GADD45B: Growth arrest and DNA damage inducible beta
GIMAP6: GTPase, IMAP family member 6
GLUL: Glutamate-ammonia ligase
HERPUD1: Homocysteine inducible ER protein with ubiquitin like domain 1
HGNC: Human genome nomenclature committee
HIF1A: Hypoxia inducible factor 1 subunit alpha
IRS2: Insulin receptor substrate 2
KEGG: Kyoto Encyclopedia of Genes and Genomes
KLF: KLF transcription factor
MDS: Multidimensionality scaling
NAMPT: Nicotinamide phosphoribosyltransferase
NFκB: Nuclear factor κB
NFKBI: NFkB inhibitor
PBMC: Peripheral blood mononuclear cell
PELI1: Pellino E3 ubiquitin protein ligase 1
PER1: Period circadian regulator 1
PFKFB3: 6-phosphofructo-2-kinase/fructose-2,6-biphosphatase 3
RBM7: RNA binding motif protein 7
RNA-seq: RNA sequencing
S1PR1: Sphingosine-1-phosphate receptor 1
SLC2A3: Solute carrier family 2 member 3
TNF: Tumor necrosis factor
TNFAIP3: TNF alpha induced protein 3
TNFSF14: TNF superfamily member 14
TSC22D3: TSC22 domain family member 3
TSS: Transcription start site
VDR: Vitamin D receptor
