# Supplementary material for: Transcriptomic profiling of immune modulation induced by vitamin D3 in the VitDPAS and VitDHiD cohort studies
Source: Sci Rep. 2025 May 19;15:17334. doi: 10.1038/s41598-025-02495-w (PMC12089289; doi:10.1038/s41598-025-02495-w)
Supplement: Supplementary file 3 — Supplementary Material 3 [file 41598_2025_2495_MOESM3_ESM.pdf]

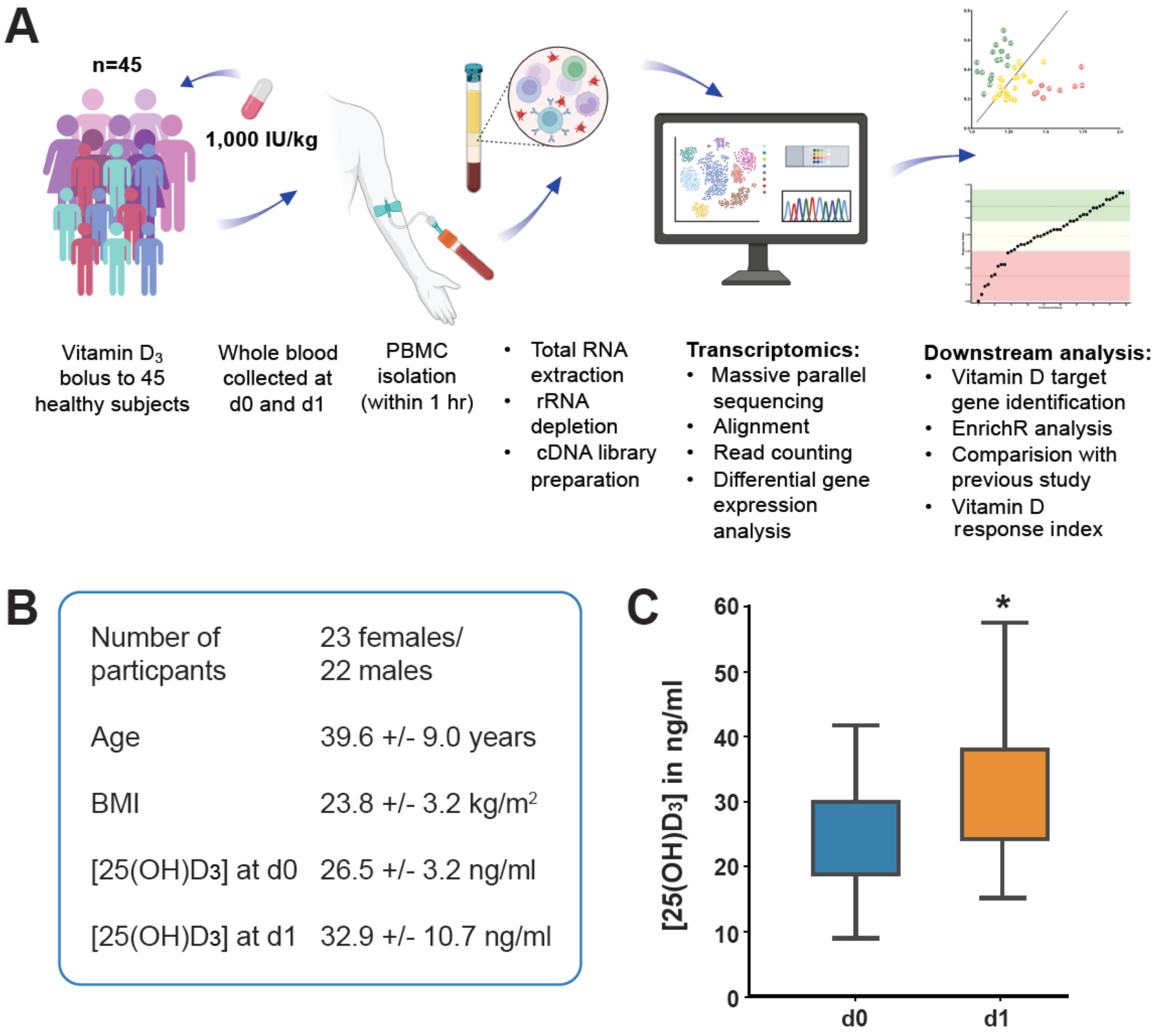

Supplementary Figure S1: Overview of the VitDPAS study. (**A**) A schematic representation of the experimental steps undertaken in the VitDPAS study. (**B**) Summary of participants' average age, BMI, and 25(OH)D_3 levels at d0 and d1 for the 45 individuals included in the study (see **Table 1** for individual-level data). (**C**) Box plots illustrating the variance and the increase in vitamin D status from d0 to d1 following a single vitamin D_3 bolus supplementation.

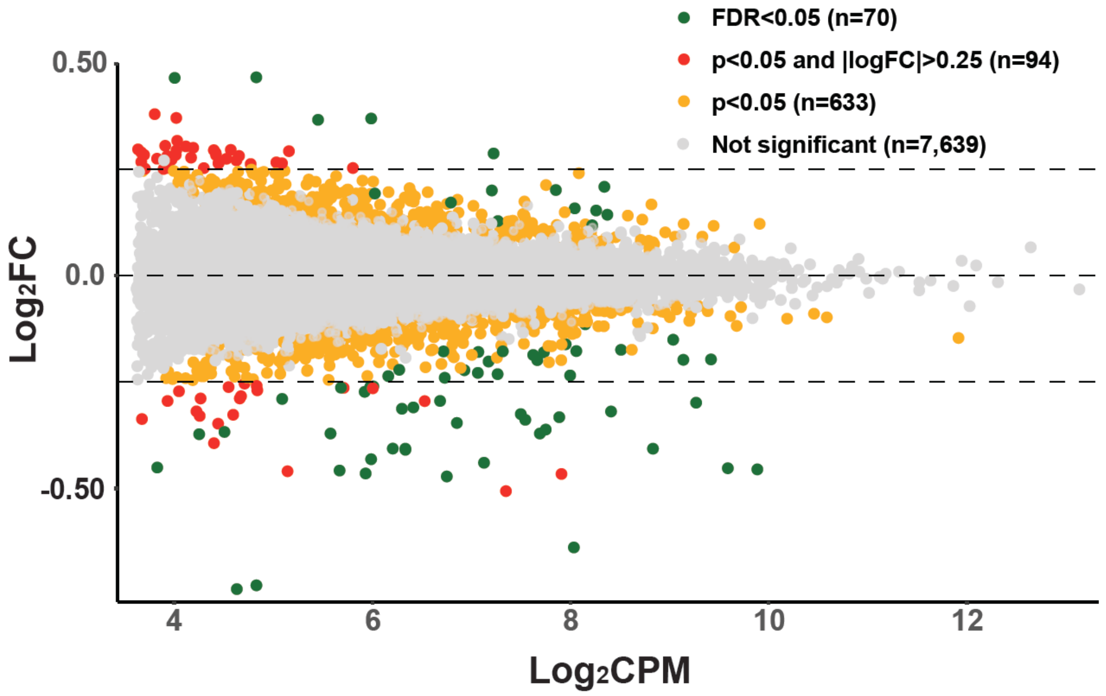

Supplementary Figure S2: Differential gene expression. A MA plot highlights the effect of vitamin D_3 bolus supplementation on the expression levels of 8,397 genes commonly expressed (CPM > 10) among all study participants.

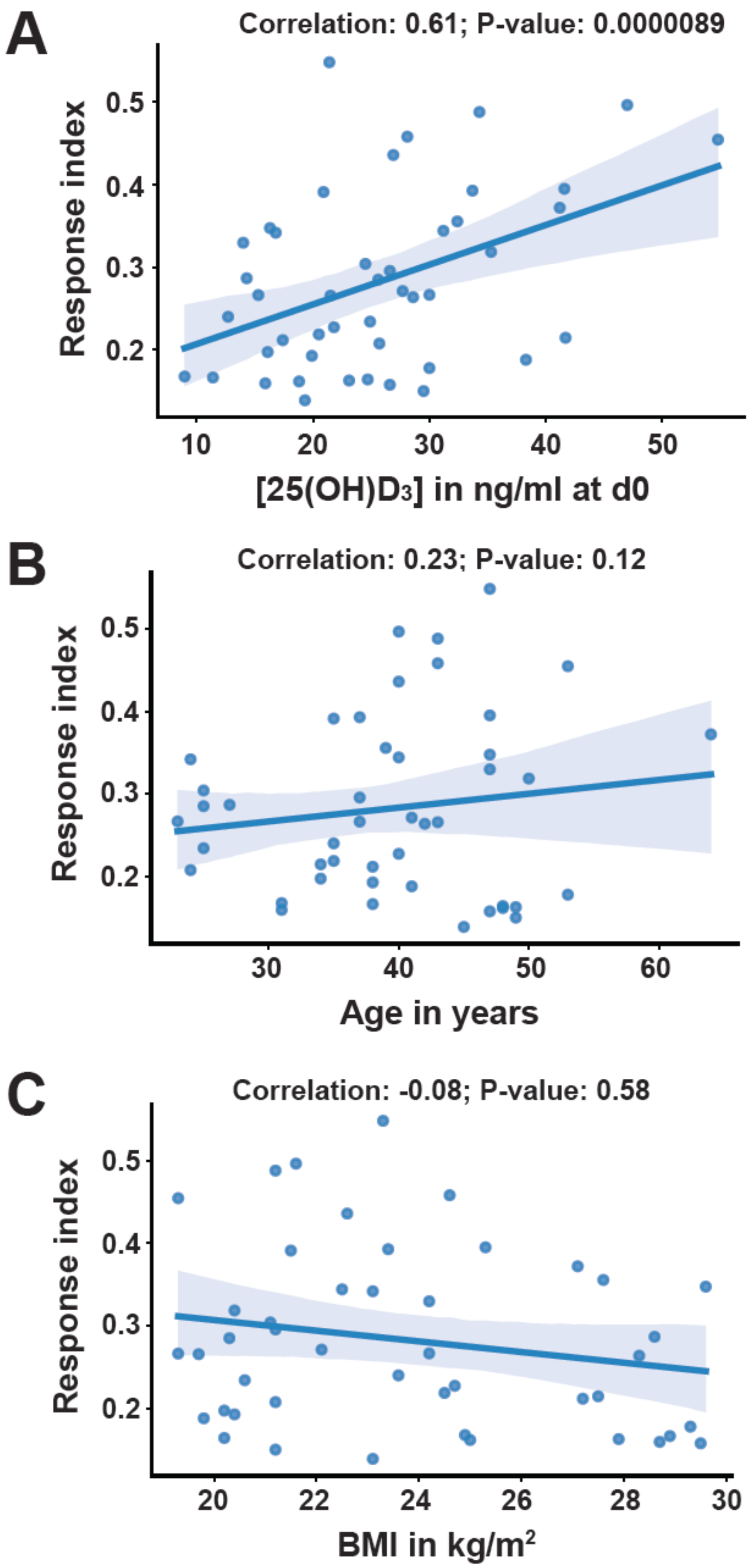

Supplementary Figure S3: Correlation of vitamin D response index with participant characteristics. The vitamin D response index for all 45 VitDPAS participants is shown in relation to 25(OH)D_3 serum levels at d0 (**A**), age (**B**), and BMI (**C**).

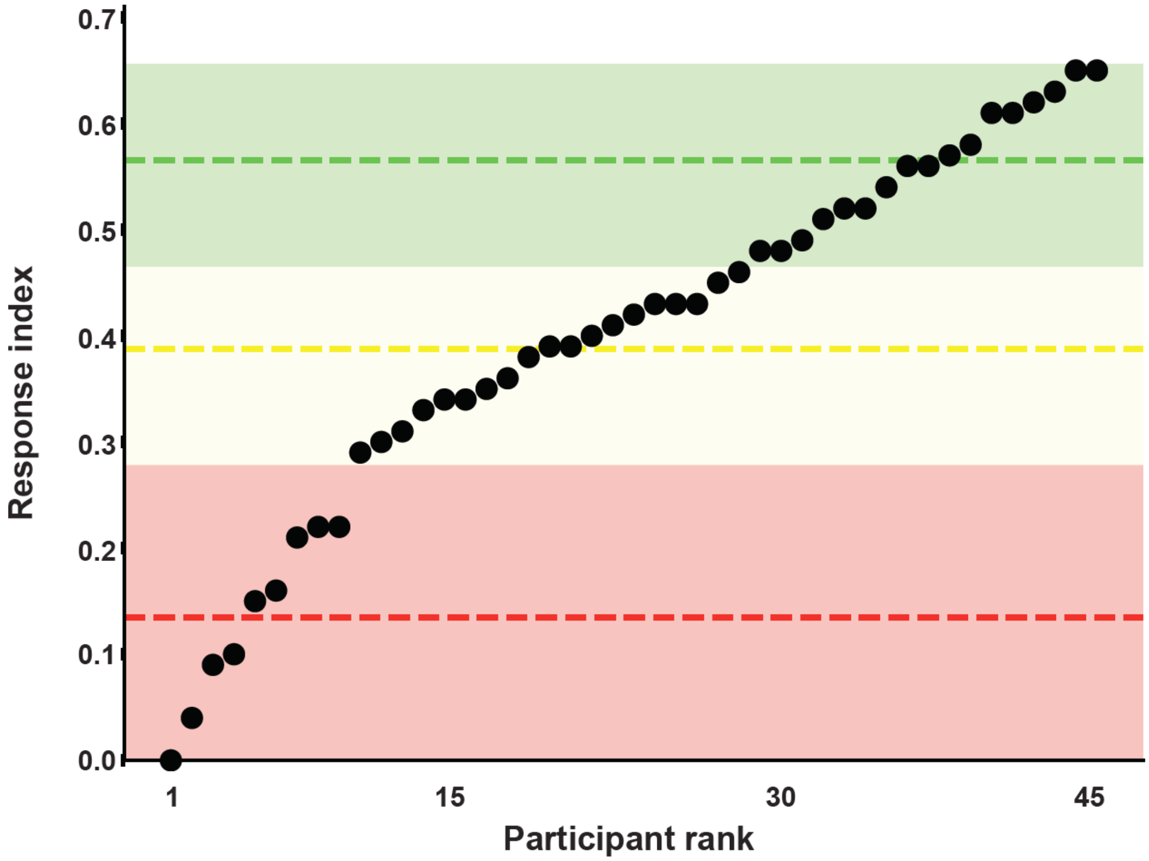

Supplementary Figure S4: Participant ranking based on vitamin D response. *K-means* clustering was applied to categorize the 45 VitDPAS study participants into low (red), mid (yellow), and high (green) vitamin D responders, based on their response index derived in **Figure 2A**.

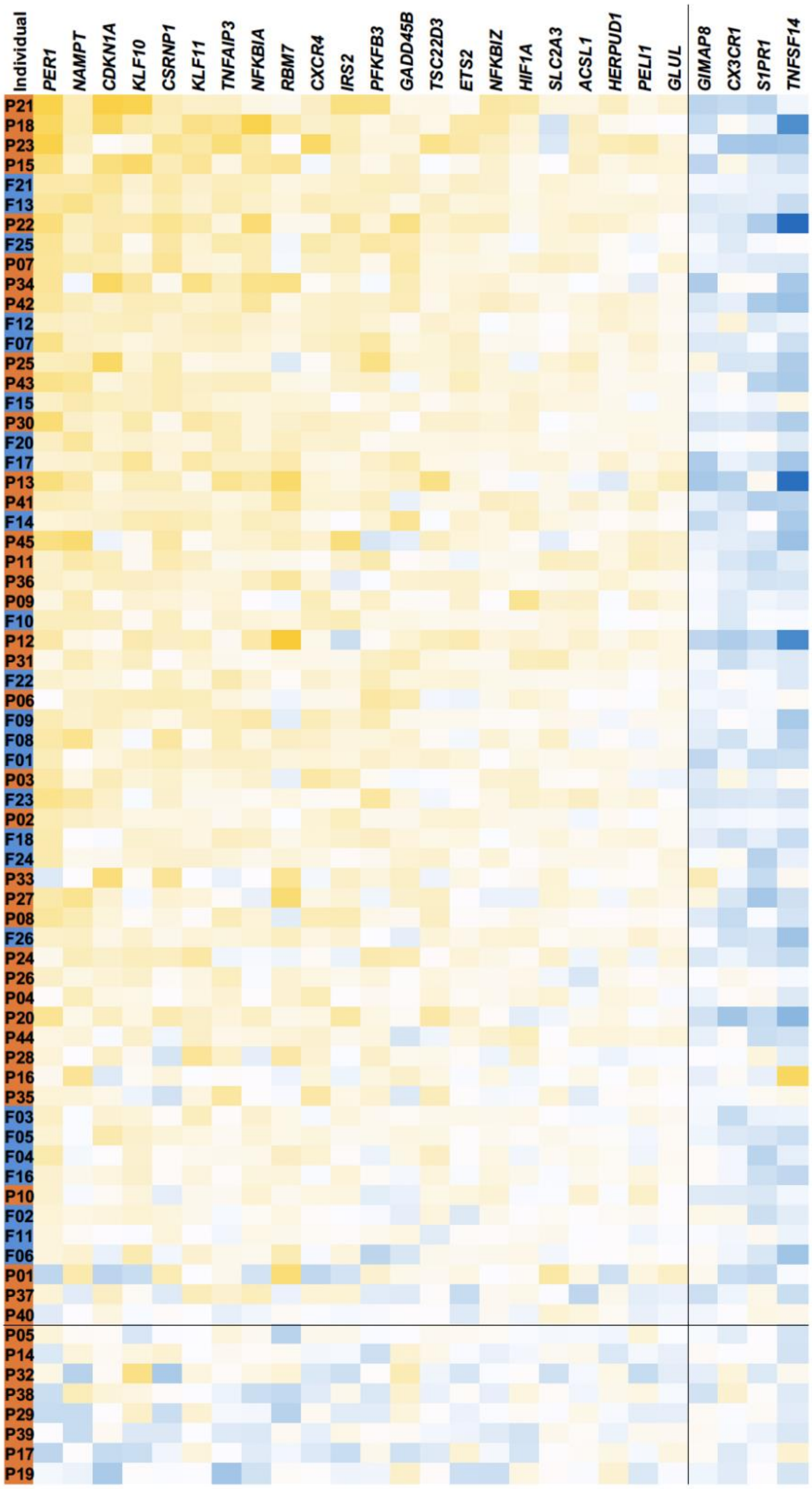

Supplementary Figure S5: Personalized regulation of common vitamin D target genes. A heatmap visualizes the regulation of 26 highly confident (FDR < 0.05) vitamin D target genes shared between the VitDPAS and VitDHiD studies. Upregulated genes are marked in blue, while downregulated genes are shown in yellow. The intensity of the color corresponds to the $\log_2$FC in gene expression between d1 and d0.

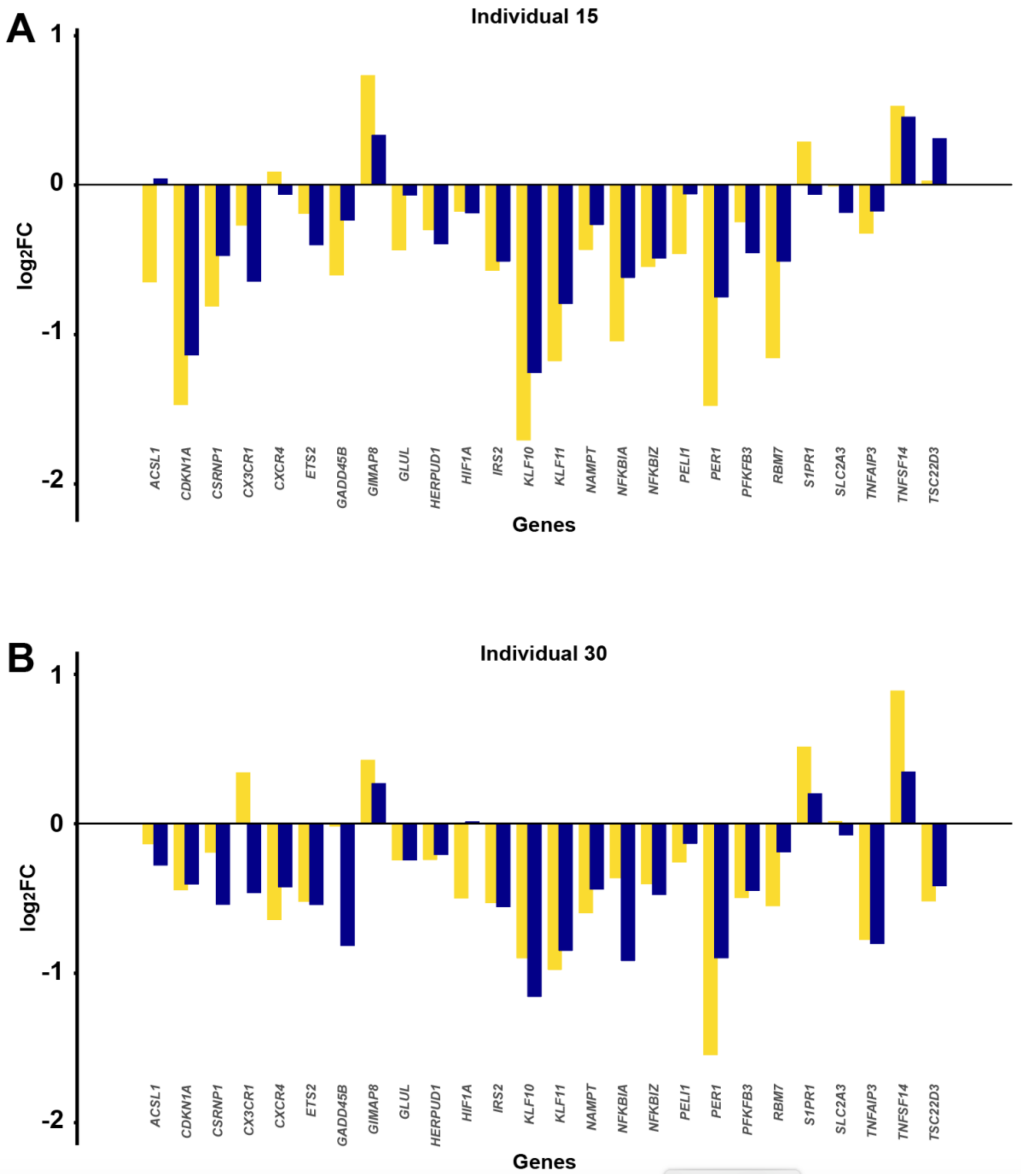

Supplementary Figure S6: Validation of key target genes using a second bolus experiment. The 26 common target genes identified between the VitDHiD study were validated through data from a repeat of the bolus experiment conducted with the same 45 participants from the VitDPAS study. Individuals 15 (**A**) and 30 (**B**) serve as representatives. The changes in RNA expression between d1 and d0 are compared for both experimental repeats. A more detailed analysis of the second bolus experiment will be presented in a separate manuscript.

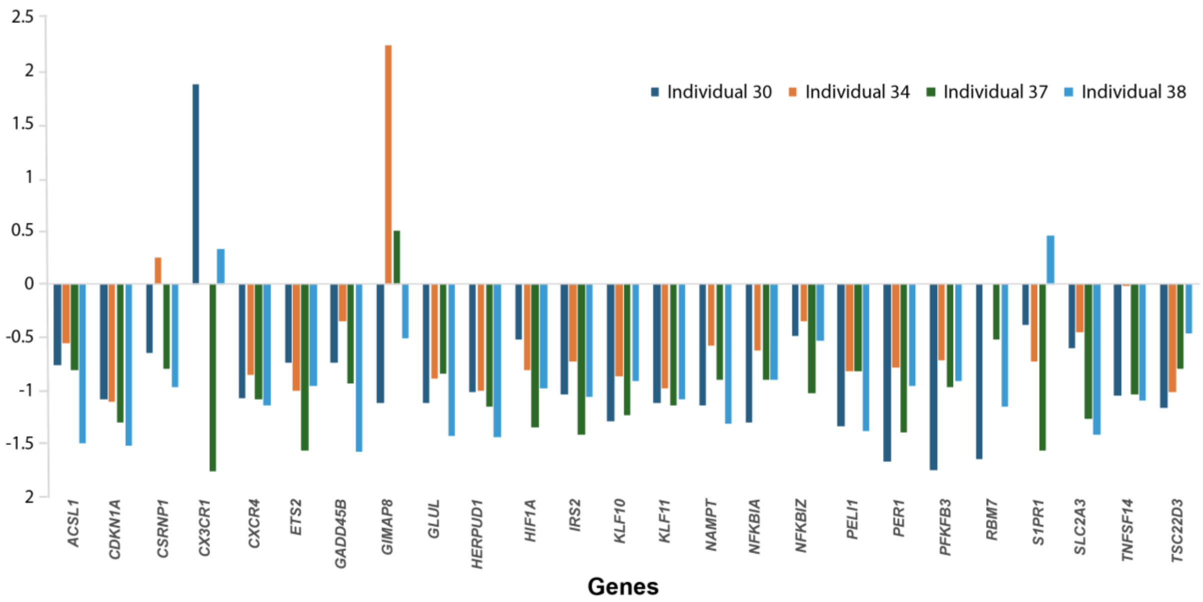

Supplementary Figure S7: Validation of key target genes through changes in chromatin accessibility at TSS regions. ATAC-seq was performed on aliquots of the same PBMC samples used in the first repeat of the VitDPAS study. Notably, no accessible chromatin was detected at the TSS region of the lowly expressed *TNFAIP3* gene. In four representative individuals, 21 genes showing downregulation at the RNA level also exhibited reduced chromatin accessibility at their TSS regions. The complete set of ATAC-seq data will be presented in a separate manuscript.

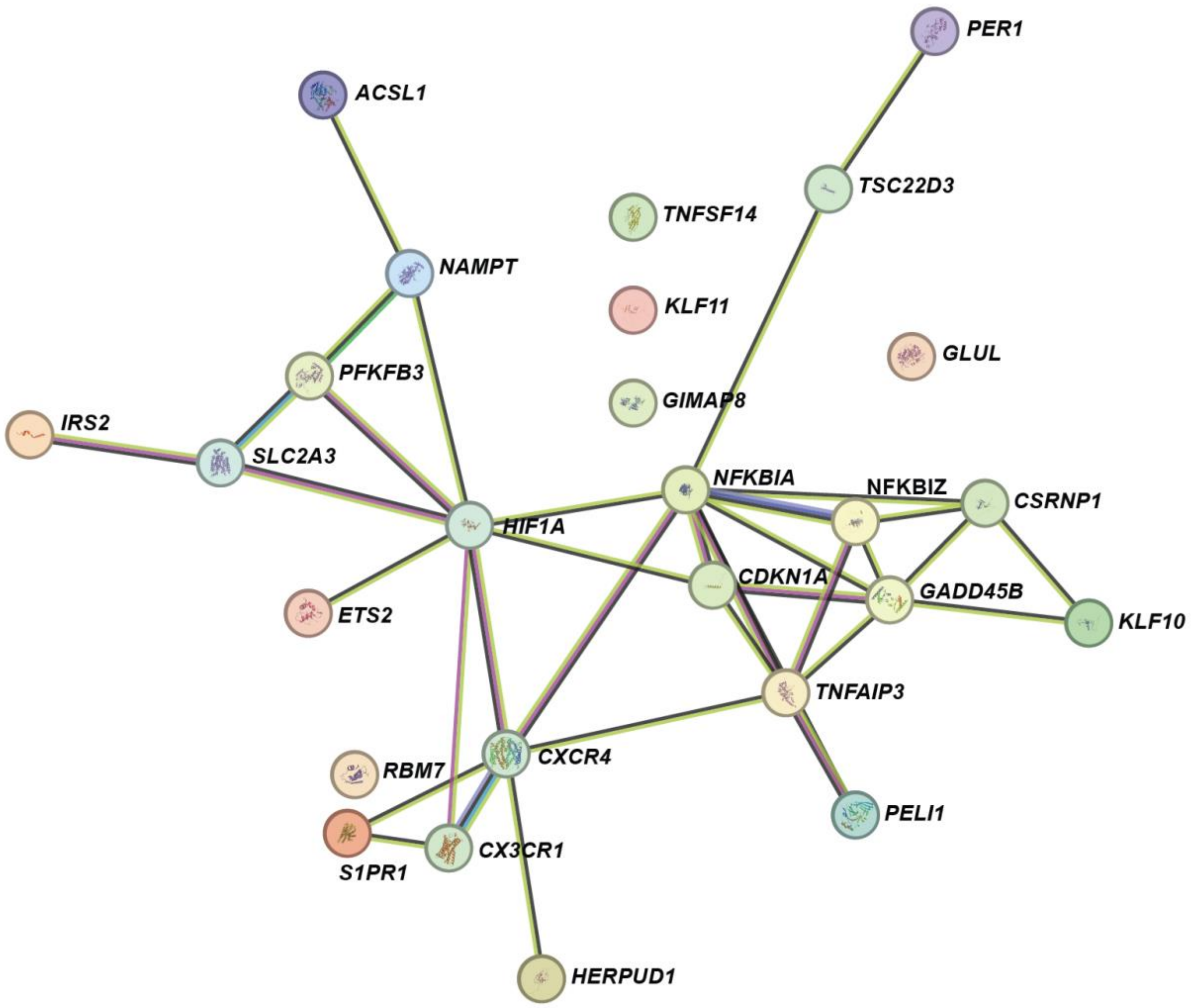

Supplementary Figure S8: Protein-protein interaction network of *in vivo* vitamin D target genes. A network diagram illustrates the protein-protein interactions among the 26 common vitamin D target genes, as visualized using the STRING database. Nodes represent proteins, while edges denote both functional and physical associations between them. The thickness of the edges reflects the strength of data supporting each interaction, providing insights into the interconnected roles of these target genes.
